# Preliminary Indications That First Semester Students From Academic Households Exhibit Higher Hair Cortisol Concentrations Than Their Peers From Nonacademic Households

**DOI:** 10.3389/fpsyt.2020.00580

**Published:** 2020-06-24

**Authors:** Alex Bertrams, Nina Minkley

**Affiliations:** ^1^Educational Psychology Lab, Institute of Educational Science, University of Bern, Bern, Switzerland; ^2^Faculty of Biology and Biotechnology, Behavioral Biology and Biology Education, Ruhr-Universität Bochum, Bochum, Germany

**Keywords:** hair cortisol concentration, parental educational background, higher education, social disparity, long-term stress

## Abstract

Previous research has extensively addressed social disparity in education using certain aspects, including the stress differences between students from nonacademic families and those from academic families during the transition from secondary school to a university. However, this issue has not yet been fully understood; the current literature suggests contradictory predictions, and physiological indicators of stress have never been assessed. Therefore, we tested whether hair cortisol concentrations (HCCs) in first semester students from nonacademic families are different from those of first semester students from academic families during their first six weeks at university. We analyzed hair samples and parental educational background reports from 71 female first semester students at a university in Switzerland in two waves (*n* = 34 in the autumn of 2016 and *n* = 37 in the autumn of 2017). The HCCs were extracted from the hair using a well-established protocol. The analyses revealed higher HCCs in the students from the academic families across the two cohorts. This difference could not be attributed to different control variables (e.g., age, migration background). These preliminary findings were in line with the sociological theory that an academic parental background is associated with pressure to avoid a drop in one's social status.

## Introduction

Social scientists have long examined the social disparities in higher education (1, 2). In order to accomplish this, researchers have often looked at the different transitional stages of educational systems. However, social disparity during the transition from a secondary school to a university is not yet fully understood.

Typically, the studies of social disparities are based on self-reports and questionnaires. However, physiological measurements may be very useful in this realm, especially regarding the measurement of stress which is a relevant variable in terms of social disparity (3–5). For the present study, we applied an interdisciplinary approach by relating the students' social status (i.e., parental educational background) to their chronic physiological stress status in the early university freshman stage.

Stress responses can be considered as “psychological, physiological, and behavioral responses to an event perceived as relevant to one's well-being with some potential for harm or loss and requiring adaptation” (6, 7). According to the transactional model of stress (7), stress emerges from an individual's perception of a threat in combination with a perceived lack of the ability to cope with it. Such negative psychological appraisals referring to stressful circumstances can be followed by a pronounced physiological cortisol response (8–11). Thereby, the hypothalamus pituitary adrenal axis is activated, which leads to the secretion of cortisol into the blood (12). The cortisol also gets into the growing hair and thus reflects the accumulated cortisol concentration over the period of hair growth (13). Therefore, a reliable and valid way to assess stress during a prolonged phase of life is by measuring the long-term cumulative hair cortisol concentration (HCC) (13). As an indication of validity, several studies have shown that serious life events (e.g., parental divorce) can lead to a significant HCC increase (14). While a temporary cortisol increase is an adaptive and mostly non-harmful response to stressors (15), chronic or long-term increased cortisol concentrations are presumed to be maladaptive and unhealthy (16).

It has been shown empirically that secondary school graduates from academic families are more likely to enroll at a university than their peers from nonacademic families with equal formal university entrance qualifications (17, 18). One crucial reason for this may be the higher motivation (or pressure) for individuals from academic households to maintain the social status of the preceding generation (17). These individuals or their families frequently perceive a decision against a university education as a social status loss for themselves and their families. Thus, they tend to enroll in a university, even in cases in which their academic abilities and expectations of success are low. In contrast, their peers from nonacademic families do not carry this burden because for them and their families careers aside from university studies do not suggest an aversive loss of social status. These theoretical considerations have been supported by empirical data [for a review, see (17)]. In contrast, Janke et al. (2) found that students from nonacademic families were somewhat more test anxious and identified less with the academic group during the first month of their first semester when compared to those from academic families. As these authors argue, undergraduates from nonacademic households may experience difficulty fitting in with the social environment at universities, and they may be more susceptible to the anticipation of academic failure.

The abovementioned theoretical thoughts and findings can lead to two contrary predictions:

1. First semester students with academic compared to nonacademic parental backgrounds exhibit higher stress levels during their beginning at university, as indicated by higher HCCs. This assumption is grounded in the increased threat of a drop in social status if one does not succeed at the university and the related social pressure (17).
2. First semester students with nonacademic compared to academic parental backgrounds exhibit higher stress levels during their beginning at university, as indicated by higher HCCs. This assumption makes sense given that students from nonacademic families are more likely to anticipate academic failure and experience test anxiety, and feel less of a sense of belonging in an academic context. This may come with an increased feeling of uncertainty to cope with academic challenges and cause problems to adjust to the new social environment (2).

Given these opposing perspectives, we tested the nondirected hypothesis that there is a difference between first semester students with nonacademic and academic parental educational background in their long-term cumulative physiological stress during their start at university. It was our intention to provide initial evidence on this matter on which more comprehensive studies can be based.

## Materials and Methods

### Participants

An *a priori* power analysis using G∗Power 3.1 (19) indicated that a sample size of at least 38 participants would be required to detect a medium-sized effect of the parental educational background (power analysis: linear multiple regression, random model, a priori; input parameters: two-tailed, H1 ρ^2^ =.30, H0 ρ^2^ = 0, α =.05, 1−β = 0.80, number of predictors = 3). We assume that considerable practical relevance is given from a medium-sized effect onwards. Considering that the potential inclusion of control variables would require a larger sample, we sampled considerably more participants.

Recruitment of participants took place in two waves (*n* = 34 in the autumn of 2016 and *n* = 37 in the autumn of 2017). Research assistants approached students who were present in various buildings of a university in the German-speaking part of Switzerland. The only criterion, whether someone should be approached or not, was a sufficient length of hair, where later the strand of hair should be cut off. Because of the length of their hair, more women were approached. If the students agreed to the question whether they were first-year students, they were invited to the study. In addition, some students were recruited *via* announcements. There were no male participants with nonacademic parental educational background in the original sample. Therefore, we excluded the four male participants with academic parental background from the analyses to avoid a potentially biasing inequality between the comparison groups [previous research has revealed differences in HCC in dependence of sex (13)].

Our final sample consisted of 71 female participants (range of age: 17–26 years). None of the participants was pregnant or breastfeeding. All participants were undergraduate students in the early freshman stage (1.5 months after beginning their university studies) who had finished secondary school in the same year. They studied a variety of subjects or subject combinations respectively.

Consistent with previous research (2, 20), the participants were classified as having nonacademic (no parent with an academic degree; *n* = 36) or academic (at least one parent with an academic degree; *n* = 35) parental backgrounds. Both the participants with non-academic and those with an academic parental educational background had obtained their school-leaving certificates (“Matura”) in different cantons (Swiss federal states). The majority of the participants had completed their Matura in the canton of Bern (*n* = 21 with nonacademic parental background, *n* = 20 with academic parental background).

### Procedure

Six weeks after their first university semester began, each of the participants provided a hair sample in order to measure their long-term stress levels *via* a HCC analysis. After providing the hair samples, the participants also filled out a questionnaire containing several self-report measures, including the measure of their parents' educational backgrounds. Finally, the participants were thanked and compensated with 20 Swiss Francs.

### Measurements

#### Hair Cortisol Concentration

Each participant provided three thin hair strands (3–5 mm thick in total). These were taken scalp near from the posterior vertex region, where the hair growth rate is most uniform (21). The 1.5 cm of hair that was next to the scalp were cut off and transferred into snap cap vials. The samples were stored at room temperature and protected from light until the cortisol concentrations could be analyzed. Assuming a hair growth rate of ∼1 cm/month (21), this hair segment represented the first 1.5 months at the university. The cortisol concentration was analyzed using the well-established protocol of Stalder and Kirschbaum (21) at the Dresden LabService GmbH laboratory in Germany.

#### Parental Educational Background

The participants indicated separately whether or not their mothers and fathers had completed academic degrees. Using these responses, we classified the participants as having nonacademic or academic parental backgrounds (for details see the sample description above).

#### Self-Reports

We collected several self-reports as potential control variables. None of these variables was of theoretical interest to us; however, their measurement allowed us to determine the comparability of the two parental educational background groups and, if necessary, to statistically control possible differences between them. For this reason, we assessed sociodemographic features (age and migration background) and basic personality traits (Big Five: openness to experience, conscientiousness, extraversion, agreeableness, neuroticism). Moreover, because these variables are typically measured in HCC research (22), we assessed self-perceived stress, hair treatments (frequency of washing hair, chemical hair treatment, and hair styling), and health and organism-related variables (body mass index, stressful events beyond starting at the university, smoking and number of cigarettes a day, momentary diseases, and momentary medication including hormonal contraceptives). In addition, we measured motivational and performance variables with regard to the educational context as these might play a role for the development of stress (study-specific self-efficacy and self-concept, self-perceived importance of studying, preparation intensity for the school leaving examination, test anxiety, school leaving examination grade). Details of the measured self-reports are given in **Table 1**. For more details, the first author will gladly send the entire questionnaire to interested persons.

**Table 1:** Overview of potential control measures (self-reports) including the descriptive statistics for the present study.

Variable	Name of measure	Number of items	Sample item	Response format	α	*M*	*SD*
Age	–	1	“Age”	Open-ended	–	19.51	1.61
Migration background	–	3	“In which country was your father born?”	Open-ended	–	–	–
Big Five - openness to experience^a^	Big Five Inventory (BFI) (23)	10	“I am someone who is original, comes up with new ideas.”	1 (not true at all) – 5 (very true)	.87	3.63	0.71
Big Five - conscientiousness^a^	Big Five Inventory (BFI) (23)	9	“I am someone who does a thorough job.”	1 (not true at all) – 5 (very true)	.83	3.57	0.63
Big Five - extraversion^a^	Big Five Inventory (BFI) (23)	8	“I am someone who is talkative.”	1 (not true at all) – 5 (very true)	.88	3.71	0.65
Big Five - agreeableness^a^	Big Five Inventory (BFI) (23)	10	“I am someone who is helpful and unselfish with others.”	1 (not true at all) – 5 (very true)	.74	3.80	0.51
Big Five - neuroticism^a^	Big Five Inventory (BFI) (23)	8	“I am someone who is depressed, blue.”	1 (not true at all) – 5 (very true)	.86	3.26	0.76
Perceived stress^a^	Perceived Stress Scale (24)	10	“Since the beginning of your studies, how often have you felt nervous and ‘stressed'?”	1 (never) – 5 (very often)	.84	2.62	0.62
Frequency of hair washing	–(22)	1	“How often do you wash your hair per week?”	Open-ended	–	4.06	1.56
Chemical hair treatment	–(22)	1	“Is your hair chemically treated? If so, how (e.g., tinting, color etc.)?”	Open-ended	–	–	–
Hair styling	–(22)	1	“Which styling method (e.g., gel, hair dryer, straightening iron) do you use frequently?”	Open-ended	–	–	–
Body height	–(22)	1	“Body height”	Open-ended	–	167.68	6.07
Body weight	–(22)	1	“Body weight”	Open-ended	–	60.46	8.30
Body mass index	–(22)	–	–	–	–	21.51	2.79
Critical and stressful life events	–(22)	2	“During the last 6 months, have you experienced something that you yourself would describe as a serious life event (e.g. the death of a close person, a serious illness or a divorce)?”	Yes – No, additional open-ended questions on what and when	–	–	–
Smoking	–(22)	1	“Are you a smoker?”	Yes – No	–	–	–
Smoking – number of cigarettes per day	–(22)	1	“If so, how many cigarettes do you smoke per day (approx.)?”	Open-ended	–	0.47	1.87
Momentary diseases	–(22)	1	“Do you currently suffer from diseases (e.g., cold, diabetes)? If so, which ones?”	Open-ended	–	–	–
Momentary medication	–(22)	1	“Are you currently taking medication (e.g., aspirin, hormonal contraception)? If so, which ones?”	Open-ended	–	–	–
Study-specific self-efficacy^a^	Study-Specific Self-Efficacy Scale (25)	6	“I feel up to the demands of studying.”	1 (does not apply at all) – 5 (completely applies)	.79	3.02	0.43
Study-specific self-concept^a^	Academic Self-Concept Scales (26)	5	“I find learning new things…”	1 (difficult) – 5 (easy)	.72	3.73	0.44
Study-specific centrality (perceived importance)^a^	–(adapted) (27)	5	“Being good at my studies means to me personally…”	1 (little) – 5 (much)	.74	4.08	0.60
School leaving examination (“Matura”): preparation intensity	–(28)	1	“How intensively have you prepared for the Matura examinations overall?”	1 (very low intensity of preparation) – 4 (very high intensity of preparation)	–	2.69	0.95
Test anxiety^a^	Test Anxiety Inventory–German (TAI–G short form) (29)	15	“I feel anxious.” (related to tests)	1 (almost never) – 4 (almost always)	.89	2.10	0.52
School leaving certification (“Matura”): grade	–	1	“Please indicate the average grade of the Matura as shown in the Matura certificate.”	4 (lowest pass grade) – 6 (excellent grade)	–	4.86	0.32

αs, Ms, and SDs are given for the overall sample (i.e., not separated by wave of measurement or parental educational background).

a Overall scores of a psychometric scale were obtained by averaging the responses to the scale items.

## Results

### Analysis Strategy

To test the hypothesis, we applied a linear multiple regression analysis regressing HCC on the data collection wave (2016 vs. 2017), parental educational background (nonacademic vs. academic), and the interaction of both predictors (data collection wave × parental educational background). Given the expected significant main effect of parental educational background, it would become clear by the coding of the variable in which of the two groups the HCC was significantly higher. A significant interaction between parental educational background and data collection wave would indicate that the expected difference between the nonacademic group and the academic group was different for the two waves (30). Prior to the analysis, we checked for outlying HCC values. Tabachnick and Fidell (31) define cases with standardized scores in excess of minus/plus 3.29 as potential outliers. In the present sample the HCC standardized scores lay between −1.99 and 2.43; thus, there were no outliers. Because the HCC lacked normality, as it is often the case in HCC studies, we log-transformed the HCC data (32).

Group differences depending on parental educational background and/or the data collection wave could provide alternative explanations for the expected main results. Therefore, we regressed each of the potential control variables on the data collection wave, parental educational background, and the interaction of both predictors. We used linear multiple regression analysis for continuous and logistic regression analysis for dichotomous potential control variables (31). In case there was at least a tendency for a main or interaction effect (i.e., *p* < .10), we considered a control variable as potentially influential. We repeated the main analysis and entered such variables in the regression model to control for their potential influence and determine whether the results of the main analysis remained constant.

### Main Analysis

**Figure 1:**
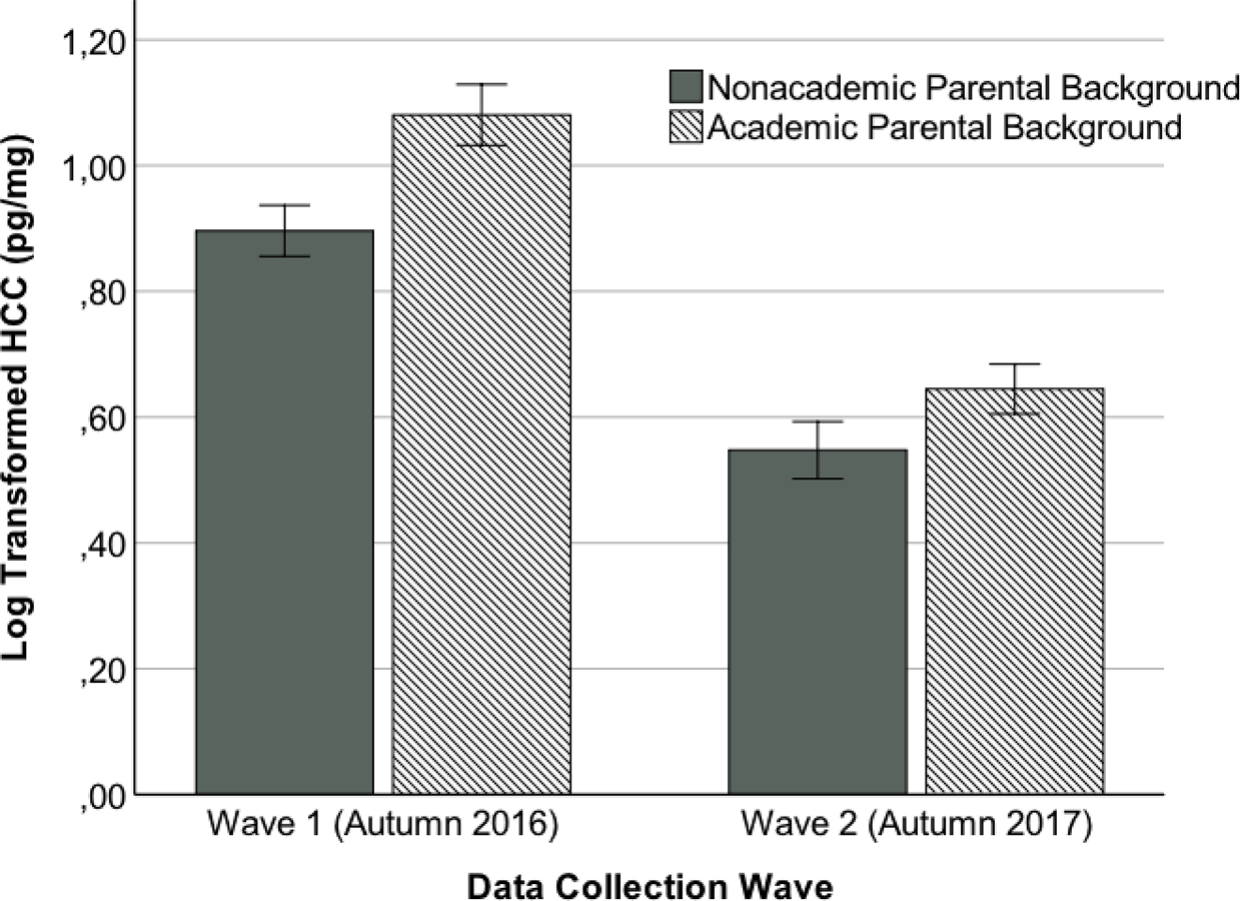
Means and standard errors for the log transformed hair cortisol concentration (HCC) as a function of the parental educational background (nonacademic vs. academic) and data collection wave (wave 1 - autumn 2016 vs. wave 2 - autumn 2017). Overall sample: *N* = 71; nonacademic parental background at wave 1: *n* = 20; academic parental background at wave 1: *n* = 14; nonacademic parental background at wave 2: *n* = 16; academic parental background at wave 2: *n* = 21.

Details on the results of the regression analysis are shown in the upper part of **Table 2**. As can be seen there, the main effects of data collection wave and parental educational background were significant. The participants with academic parental backgrounds displayed higher cortisol concentrations (*M* = 0.82 pg/mg, *SD* = 0.27 pg/mg) than the participants with nonacademic parental backgrounds (*M* = 0.74 pg/mg, *SD* = 0.26 pg/mg). Moreover, the HCCs assessed in 2016 (*M* = 0.97 pg/mg, *SD* = 0.22 pg/mg) were generally higher than the HCCs assessed in 2017 (*M* = 0.60 pg/mg, *S*D = 0.16 pg/mg). The interaction between the data collection wave and the parental educational background was nonsignificant. **Figure 1** illustrates these results.

**Table 2:** Multiple regression analysis regressing hair cortisol concentration.

**1) Without control variables (main analysis)**						
**Predictor**	***B***	***B*** **95% CI**	***SE B***	**β**	***t***	***p***
Constant	0.90	[0.82, 0.98]	0.04	–	22.13	<.001
Data collection wave	−0.35	[−0.47, −0.23]	0.06	–.66	–5.74	<.001
Parental educational background	0.18	[0.06, 0.31]	0.06	.35	2.92	.005
Data collection wave × parental educational background	−0.09	[−0.26, 0.09]	0.09	−.15	−1.00	.32
**2) Including control variables (supplementary analysis)**						
**Predictor**	***B***	***B*** **95% CI**	***SE B***	**β**	***t***	***p***
Constant	1.12	[0.41, 1.83]	0.35	–	3.17	.002
Data collection wave	−0.32	[−0.46, −0.18]	0.07	−.60	−4.47	<.001
Parental educational background	0.18	[0.04, 0.31]	0.07	.33	2.51	.02
Data collection wave × parental educational background	−0.12	[−0.31, 0.08]	0.10	−.21	−1.23	.22
Age	−0.03	[−0.06, 0.01]	0.02	−.15	−1.64	.11
Migration background	−0.02	[−0.14, 0.10]	0.06	−.03	−0.38	.71
Big Five openness	0.04	[−0.03, 0.10]	0.03	.09	1.02	.31
Perceived stress	0.01	[−0.07, 0.08]	0.04	.01	0.13	.90
Frequency of hair washing	−0.01	[−0.04, 0.03]	0.02	−.04	−0.43	.67
Centrality (importance)	0.02	[−0.07, 0.10]	0.04	.04	0.45	.66
Matura preparation intensity	0.03	[−0.02, 0.08]	0.03	.10	1.07	.29

N = 71. Coding of the data collection wave: 0, wave 1; 1, wave 2. Coding of the parental educational background: 0, nonacademic; 1, academic.

### Supplementary Analysis

As **Table 3** shows, there were tendentious differences for the following variables: age, migration background, Big Five openness, perceived stress since the beginning of studying, frequency of hair washing, study-specific centrality, and Matura preparation intensity. However, when we entered these variables as additional predictors in the multiple regression analysis, the main results did not change (see **Table 2**, lower part). In this regression model none of the control variables was a significant predictor of HCC.

**Table 3:** Group comparisons for potential control variables.

1) Regressing continuous variables (linear multiple regression analyses)										
Variable	Data collection wave 1				Data collection wave 2				Significance tests (*p*)		
	**Nonacademic** **PEB**		**Academic** **PEB**		**Nonacademic** **PEB**		**Academic** **PEB**				
	*M*	*SD*	*M*	*SD*	*M*	*SD*	*M*	*SD*	Main effect wave	Main effect PEB	Wave × PEB
HCC (pg/mg; logarithmized)	0.90	0.22	1.08	0.19	0.55	0.17	0.64	0.15	< .001	.005	.32
Age	19.05	1.28	19.50	1.02	20.56	2.31	19.14	1.28	.004	.40	.01
Big Five openness	3.46	0.78	4.06	0.51	3.32	0.74	3.76	0.59	.55	.01	.60
Big Five conscientiousness	3.62	0.50	3.52	0.55	3.56	0.74	3.57	0.73	.76	.66	.71
Big Five extraversion	3.83	0.65	3.76	0.71	3.79	0.54	3.52	0.69	.85	.75	.53
Big Five agreeableness	3.91	0.56	3.63	0.50	3.94	0.44	3.70	0.51	.84	.12	.86
Big Five neuroticism	3.07	0.71	3.21	0.83	3.31	0.78	3.42	0.74	.34	.58	.91
Perceived stress	2.37	0.52	2.72	0.64	2.76	0.68	2.70	0.62	.06	.10	.17
Frequency of hair washing	3.90	1.29	3.68	1.54	5.19	1.59	3.62	1.47	.01	.67	.06
Body height (cm)	166.15	5.91	166.43	4.97	168.75	5.93	169.14	6.83	.20	.90	.97
Body weight (kg)	60.00	8.35	62.79	10.49	61.19	7.61	58.80	7.27	.67	.34	.20
Body mass index	21.73	2.86	22.71	3.87	21.48	2.33	20.52	1.93	.79	.31	.15
Number of cigarettes per day	0	0	0.59	1.45	0.94	3.11	0.48	1.78	.14	.37	.25
Self-efficacy	3.15	0.44	3.02	0.42	3.00	0.32	2.91	0.48	.30	.40	.85
Self-concept	3.78	0.27	3.83	0.60	3.60	0.39	3.71	0.48	.23	.75	.76
Centrality (importance)	3.95	0.62	3.79	0.45	4.41	0.49	4.14	0.64	.02	.41	.70
Matura preparation intensity	2.50	0.89	2.64	1.15	3.06	0.77	2.62	0.97	.08	.67	.20
Test anxiety	2.01	0.38	2.04	0.65	2.12	0.46	2.22	0.58	.54	.89	.75
Matura grade	4.82	0.27	4.89	0.25	4.89	0.41	4.85	0.34	.49	.51	.46

**2) Regressing dichotomous variables (logistic regression analyses)**											
**Variable**	**Data collection wave 1**				**Data collection wave 2**				**Significance tests (*p*)**		
	**Nonacademic** **PEB**		**Academic** **PEB**		**Nonacademic** **PEB**		**Academic** **PEB**				
	**Yes (*n*)**	**No (*n*)**	**Yes (*n*)**	**No (*n*)**	**Yes (*n*)**	**No (*n*)**	**Yes (*n*)**	**No (*n*)**	**Main effect wave**	**Main effect PEB**	**Wave × PEB**

Migration background	1	19	4	10	3	13	6	15	.22	.09	.30
Any chemical hair treatment	8	12	7	7	5	11	11	10	.59	.56	.63
Any hair styling	15	5	9	5	12	4	17	4	1.00	.50	.44
Critical/stressful life events^a^	12	8	9	5	6	10	15	6	.18	.80	.22
Smoking	0	20	3	11	3	13	3	18	.998	.998	.998
Any momentary diseases	4	16	3	11	3	13	2	19	.93	.92	.50
Any momentary medication^b^	8	12	7	7	9	7	12	9	.33	.56	.70
Any contraceptive use	6	14	6	8	9	7	11	10	.12	.44	.47

N = 71. Coding of the data collection wave: 0, wave 1; 1, wave 2. Coding of the parental educational background: 0, nonacademic; 1, academic. HCC, hair cortisol concentration; PEB, parental educational background.

a Statements about stress with direct regard to studying were not coded as stress here.

b This includes hormonal contraceptives.

## Discussion

The present study revealed preliminary evidence that, on average, female first semester students from academic households exhibited higher physiological long-term stress (HCC) during the initial six weeks at the university than their peers from the nonacademic families. In other relevant characteristics the two groups did not differ or the control of (tendentious) differences in the analyses did not change the results. One implication of these findings is that, relative to the first semester students from nonacademic households, those from academic households are supposed to be at a higher risk for developing psychosocial and physical pathological conditions (16). This may still be the case although these two groups did not differ in self-perceived stress. Self-perceptions of stress are frequently unrelated to cortisol responses (33); thus, they seem to represent a conceptually different variable.

Our findings are in line with the theoretical and empirical work of sociologists who argue that individuals from academic families may be frightened of experiencing a social (academic) drop if they fail while attending a university (17). Based on this argumentation, we assumed that there may be social pressure for academic family members to continue the family tradition, that is, to acquire a higher education at a university. This pressure may cause them to enroll in academic studies, even in cases in which their motivation or ability are insufficient. Low motivation and/or ability may shape the perception of the transition from school to university as threatening, and the possibility of coping with the threat as low, thereby increasing the stress (7). The outlined situation of students from academic households can also be considered as strong social-evaluative threat. As described in the meta-analysis of Dickerson and Kemeny (34), tasks comprising social-evaluative threat and uncontrollability were associated with the largest cortisol increases, which would be displayed in higher HCCs.

However, another explanation for the present findings could be that individuals from nonacademic families are particularly unstressed because they (and their families, respectively) can only gain (but not lose) an academic status. Thus, in a sense, they cannot “fall down” with respect to this aspect of societal structures. Both groups reported an equally high subjective importance of their studies. Possibly this importance is fed by different motives (fear of social decline vs. hope of social rise).

The present study has some limitations. The present findings must be regarded as preliminary, as they relate to a particular group of women in Switzerland (i.e., undergraduates at one Swiss university). Thus, the findings must not be overgeneralized to all female undergraduates. Future and more comprehensive research should provide a more representative picture, and the present initial results suggest that a more elaborate study might be worthwhile. Such further research should also take into account possible differences between different study programs. We were not able to analyze such differences as we did not have sufficient data available. Moreover, it should be noted that our theoretical considerations on the psychological processes underlying the present results are speculative, as we have not collected data on this. Further research is also needed in this respect. In addition, the present study did not provide insight into what factors caused higher HCCs in 2016 equally in both groups of first semester students. We cannot enlighten this at this point. It is possible that certain societal developments or environmental conditions had a general influence on HCC. However, such considerations cannot go beyond pure speculation.

Turning to a strength of the present work, we are not aware of any other study that has applied a neuroendocrine measure of stress to the extensively and internationally investigated issue of social disparity in higher university education (in the vast majority, self-reports were used). In contrast, the usefulness of biological measures (such as hormone concentrations) to examine certain aspects of social disparity in childhood has been demonstrated in the past (35). Thus, the present findings may be a promising basis for further research using natural science methods to investigate the aspects of social disparity in higher education.

## Conclusion

Our preliminary findings suggest that whether students come from a nonacademic or academic home may be relevant for their physiological long-term stress at the beginning of their university studies. The present study provides a basis for more detailed further research on parental educational backgrounds and physiological stress measures.

## Data Availability Statement

The datasets generated for this study are available on request to the corresponding author.

## Ethics Statement

The studies involving human participants were reviewed and approved by the ethics committee of the Faculty of Human Sciences at the University of Bern. The patients/participants provided their written informed consent to participate in this study.

## Author Contributions

AB conceived the project, designed the study, conducted the experiment, analyzed the data, and wrote the manuscript. NM conceived the project, designed the study, analyzed the data, and wrote the manuscript.

## Conflict of Interest

The authors declare that the research was conducted in the absence of any commercial or financial relationships that could be construed as a potential conflict of interest.
